# Evaluation of efficacy of Interceptor^®^ G2, a long-lasting insecticide net coated with a mixture of chlorfenapyr and alpha-cypermethrin, against pyrethroid resistant *Anopheles gambiae* s.l. in Burkina Faso

**DOI:** 10.1186/s12936-017-1846-4

**Published:** 2017-05-08

**Authors:** Koama Bayili, Severin N’do, Moussa Namountougou, Roger Sanou, Abdoulaye Ouattara, Roch K. Dabiré, Anicet G. Ouédraogo, David Malone, Abdoulaye Diabaté

**Affiliations:** 1 Intitut de Recherche en Sciences de la Santé/Centre Muraz, Bobo-Dioulasso, Burkina Faso; 2 Universite Polytechnique de Bobo, Bobo-Dioulasso, Burkina Faso; 3 Innovative Vector Control Consortium/Liverpool, Liverpool, UK

**Keywords:** Malaria, *Anopheles gambiae*, Chlorfenapyr, Insecticide, Bed net, Pyrethroid resistance

## Abstract

**Background:**

Malaria vectors have acquired widespread resistance throughout sub-Saharan Africa to many of the currently used insecticides. Hence, there is an urgent need to develop alternative strategies including the development of new insecticides for effective management of insecticide resistance. To maintain progress against malaria, it is necessary to identify other residual insecticides for mosquito nets. In the present WHOPES phase II analogue study, the utility of chlorfenapyr, a pyrrole class insecticide mixed with alpha-cypermethrin on a long-lasting mosquito bed net was evaluated against *Anopheles gambiae* s.l.

**Methods:**

Bed nets treated with chlorfenapyr and alpha-cypermethrin and mixture of both compounds were tested for their efficacy on mosquitoes. Washed (20 times) and unwashed of each type of treated nets and were tested according to WHOPES guidelines. Efficacy of nets were expressed in terms of blood-feeding inhibition rate, deterrence, induced exophily and mortality rate. The evaluation was conducted in experimental huts of Vallée du Kou seven (VK7) in Burkina Faso (West Africa) following WHOPES phase II guidelines. In addition, a WHOPES phase I evaluation was also performed.

**Results:**

Mixture treated nets killed significantly (P < 0.05) more mosquitoes than solo alpha-cypermethrin nets, unwashed and washed. Proportionally, this equated to mortalities of 78 and 76% (for mixture nets) compared to only 17 and 10% (for solo alpha-cypermethrin) to *An. gambiae*, respectively. In contrast mixture net proportions were not significantly (P > 0.05) different from nets treated with chlorfenapyr 200 mg/m^2^ unwashed (86%). The washed and unwashed nets treated with the mixtures resulted in personal protection against *An. gambiae* s.l. biting 34 and 44%. In contrast the personal protection observed for washed and unwashed alpha-cypermethrin treated nets generated (14 and 24%), and chlorfenapyr solo treated net was rather low (22%).

**Conclusion:**

Among all nets trialled, the combination of chlorfenapyr and alpha-cypermethrin on bed nets provided better mortality in phase II after 20 washes. Results suggest that this combination could be a potential insecticide resistance management tool for preventing malaria transmission in areas compromised by the spread of pyrethroid resistance.

## Background

Malaria remains a serious public health issue although significant reduction in the disease burden has been observed over the last few years. The large-scale implementation of long-lasting insecticide-treated nets (LLIN and indoor residual spraying (IRS) have played a major role in this battle against malaria. Several studies have demonstrated the efficacy of both tools in curbing malaria incidence [1–3]. During the last decade, the massive roll out of LNs has allowed a significant reduction in malaria-associated morbidity and mortality across sub-Saharan Africa [4, 5]. Pyrethroids remain ideal insecticides for treating nets owing to their low cost, longer residual activity and safety [4, 6, 7]. However, the emergence and subsequent spread of insecticide resistance in major mosquito vector species could jeopardize the success of malaria control programmes [8, 9] relying on this mode of action to control them. Resistance to pyrethroids has now been reported in all major malaria vectors in 27 countries across sub-Saharan Africa [5, 10].

In Burkina Faso, insecticide resistance in mosquito vector populations appeared as early as the 1960s when *Anopheles funestus* and *Anopheles gambiae* s.l. populations were demonstrated to have reduced mortality to dieldrin and DDT [7, 11]. This resistance is quickly spreading across the country and has now been reported in the *An. gambiae* species complex [12–15]. Recently, it has been demonstrated that vector species *An. gambiae s.l.* has developed high level resistance to pyrethroids and other classes of insecticides [16]. Resistance through a combination of L1014F *kdr* and CYP6P3P450 mechanisms and other metabolic enzymes were present in VK7 species, including two carboxylesterases (COEAE3G, COEAE4G) and a GST (GSTE5) [18].

In Benin, a country close to Burkina Faso, the situation has become alarming in the southern part of the country where the *An. gambiae* complex is reported resistant to pyrethroid LNs, and has demonstrated only limited personal protection that inadequately kill mosquitoes [17]. Similarly, in Burkina Faso, a recent study has shown that resistance to pyrethroids has increased by more than 1000-fold over the last few years [18]. These examples underscore the urgent need for alternative tools or new insecticide formulations to complement existing ones and preserve LNs effectiveness. In the short term, novel or repurposed insecticide classes with dissimilar modes of action could be used either alone or in combination with pyrethroids for IRS and bed nets.

Chlorfenapyr, a pyrrole insecticide (IRAC group 13) with a completely different mode of action, could be a viable alternative to pyrethroids [19, 20]. Recent studies in experimental huts have shown that chlorfenapyr was more effective on resistant *Anopheles* and *Culex* populations than pyrethroids [21, 22]. As chlorfenapyr acts as a metabolic toxin, it does not show the repellency and the knock down typical for neurotoxins like the pyrethroids. Repellency is crucial for reducing mosquito biting rates and providing personal protection to net users. Thus, the combination of chlorfenapyr with a pyrethroid should enhance LNs users’ protection and afford avoidance or reduction of resistance selection. This solo pyrethroid LNs have been proposed for these challenges. The combination of chlorfenapyr applied as IRS with LNs increased the protection against mosquito bites and enhanced the control of the disease transmission [23]. More recently, studies carried out on mixtures of chlorfenapyr and pyrethroids (alpha-cypermethrin and permethrin) have shown effective control of resistant populations of *An. gambiae* and *Culex quinquefasciatus* [24, 25]. In Burkina Faso, malaria vectors have posited selection and resistance to all available classes of insecticides in the vicinity to the rice growing area of Vallée du Kou, while also increasing their resistance intensity over 1000-fold in the last few years, thus threatening the future of pyrethroid LNs [18]. The magnitude of pyrethroid resistance and the multi-mechanisms developed by mosquitoes, make this specific ecological setting an ideal place to test efficacy of new insecticides or new formulations. Indeed, it has been recently demonstrated that new LNs of different brands had almost no killing effect on field collected mosquitoes [26].

The objective of this study was to assess the efficacy of Interceptor^®^ G2, an LN with a mixture of chlorfenapyr and alpha-cypermethrin, in an area where pyrethroid nets have limited efficacy to known mosquito malaria vectors. This study will be among the first evaluations of a LN with two discrete insecticides with completely different modes of action that provide some indication of their suitability to control wild insecticide resistant mosquitoes.

## Methods

### Study site

The study was carried out in Vallée du Kou 7 (VK7), a permanent source of irrigation with breeding sites which are preferentially colonized by *Anopheles coluzzii* although *An. gambiae* also is found [13]. The site is characterized by wooded savannah and covers 1200 ha between 4°24′W and 11°24′N. Resistance to DDT and pyrethroids is widespread in both mosquito species. Resistance to these classes of insecticides is due to the *kdr* mutation which is almost fixed in the populations [16] and to detoxifying enzymes [26].

### Mosquito net treatments and trial procedure

#### Design of huts

The experimental huts are made from concrete bricks, with a corrugated tin roof, a ceiling of thick polyethylene sheeting, and a concrete base surrounded by a water-filled channel to prevent entry of ants following the WHO Guidelines for laboratory and field-testing of long-lasting nets [29] where illustrations of the design of a West-African style hut can be found. Mosquito access occurs via 4 window slits constructed from pieces of metal, fixed at an angle to create a funnel with a 1-cm wide gap. Mosquitoes fly upward to enter through the gap and then downwards to exit into the hut; this precludes or greatly limits exodus though the aperture enabling most of the entering mosquitoes to be accounted for. A single veranda trap made of polyethylene sheeting and screening mesh measuring 2-m long, 1.5-m wide, and 1.5-m high, projects from the back wall of each hut. Movement of mosquitoes between hut and veranda is unimpeded during the night.

#### Insecticide treatments

The nets were made of 100-denier polyester, factory-coated with formulations of alpha-cypermethrin and chlorfenapyr (BASF, Germany). Six standardized holes (each measuring 4 cm × 4 cm) were cut into the sides (two on the length and one on the width) of each net as recommended by WHO [27] to simulate torn nets. Nets were washed individually in 10 L of spring water containing 2 g/L of soap (Savon de Marseille), at 20 rotations per minute during 10 min immersion and then rinsed twice. Interceptor^®^ G2 LN unwashed and 20 times washed were tested according to WHOPES guidelines, against an untreated net (control) and a reference WHOPES-approved net (Interceptor^®^) [28]. The described washing procedure is a standardized process outlined by WHOPES to simulate use nets in the real world, but comparable conditions per WHOPES phase II guidelines [28].

The following treatments were tested: Interceptor^®^ G2 LN, Interceptor^®^ LN and chlorfenapyr formulation (Phantom 240 g/L SC) were supplied by BASF SE. The target concentrations were 100 mg/m^2^ alpha-cypermethin and 200 mg/m^2^ chlorfenapyr on Interceptor^®^ G2 LN, 200 mg/m^2^ alpha-cypermethrin on Interceptor^®^ LN and 200 mg/m^2^ on the chlorfenapyr solo net. All nets were 100 denier polyester. The following 6 treatment arms were compared in the experimental huts: Interceptor^®^ G2 LN unwashed, washed 20 times; Interceptor^®^ LN unwashed, washed 20 times; chlorfenapyr hand-treated net and untreated net.

#### Sleepers and mosquito collection

Adult volunteers slept under the nets and mosquitoes were collected the next morning. The volunteers were recruited among the inhabitants of the study site after reading and signing the informed consent. The treatments were randomly allocated to 6 experimental huts and treatments rotated weekly between huts while sleepers rotated on consecutive nights to adjust for any difference in hut and individual attractiveness. Volunteers started collecting mosquitoes in the nets and in the different compartments of the huts at 5:30 a.m. Mosquitoes were brought to the laboratory of Intitut de Recherche en Sciences de la Santé (IRSS), Centre Muraz, Bobo-Dioulasso, Burkina Faso, for species identification, mortality counts and determination of blood-feeding status. Living mosquitoes were put in small cups netted with plastic and provided 10% sugar solution under laboratory conditions (27 ± 2 °C and 80 ± 10% RH) for 72 h to assess delayed mortality. Mosquitoes were collected over eight weeks between August and October 2014. The following outcomes were measured: (i) deterrence (reduction in hut entry relative to the control huts fitted with untreated nets); (ii) induced exophily (the proportion of mosquitoes found in exit traps relative to the total collected mosquitoes); (iii) blood-feeding inhibition (the reduction in blood-feeding of mosquitoes relative to the control huts); (iv) immediate and delayed mortality (the proportion of dead mosquitoes when collected and after 24 h); (v) personal protection: the reduction in mosquito biting by treated nets relative to untreated nets, as derived from the formula:  % personal protection = 100× (Bu − Bt/Bu). Where Bu is the total number blood-fed mosquitoes in the huts with untreated nets and Bt is the total number blood-fed in the huts with treated nets.

Field efficacy was compared to untreated control nets and a commercial standard Interceptor^®^ net approved by WHOPES. A chlorfenapyr dipped net was also used for comparison in experimental hut trials for reference.

#### Ethical clearance

Written informed consent was obtained from all volunteers recruited in this study. The study was approved by the institutional ethic committee of IRSS/Centre Muraz (015-2014 CE-CM).

### *In situ* bioassay

*Anopheles gambiae* s.l. larvae were collected in August and September 2014 from natural breeding sites in the study area. Larvae were brought to the insectary at IRSS in Bobo-Dioulasso where they were fed Tetramin™ baby fish food and reared to adults under standard controlled conditions (27 ± 2 °C, 80 ± 10% RH and 12:12 L–D). Upon emergence, adults were maintained on 5% sugar solution and used for various tests. The Kisumu strain of *An. gambiae,* an insecticide-susceptible strain, was also reared simultaneously under the same condition as the field-collected larval and was used as a control for bioassay.

#### Cone test

Cone testing was performed at the beginning and at the end of the trial. For each net, 10 cones were placed on five sections of the net (4 sides and the roof). Larvae of *An. gambiae* s.l. were collected from the field and brought to the insectary of IRSS for rearing. At emergence, 10 unfed females aged 3–5 days were introduced in each cone and exposed to the nets for 30 min. In total 100 mosquitoes were used per net. After exposure, the mosquitoes were provided 10% sugar meal and brought back to the insectary for mortality recording at 24 and 72 h. Mosquitoes were kept under the same laboratory conditions described above. The insecticide-susceptible strain of *An. gambiae* Kisumu was used as control.

#### Chemical analysis

Netting samples were taken for determination of alpha-cypermethrin and chlorfenapyr content by gas chromatographic (GC) on two occasions: before washing and after conclusion of the trial, as described by WHO [29]. Five net pieces, each measuring 30 cm × 30 cm, were cut from the sides end and the top of the nets and kept in aluminium foil at 4 °C in the refrigerator. A total of 100 net pieces were sent to BASF for the chemical contents analysis. The gas chromatographic (GC) analysis was carried out on each piece applying CIPAC 454/LN method for both alpha-cypermethrin and chlorfenapyr.

### Laboratory study

#### Tunnel test

The tunnel test measures mortality and blood-feeding success of host seeking mosquitoes in an experimental chamber. This experiment was designed to provide further insight and explanation of the toxicity of unwashed and washed nets used in the huts. Pieces of nets cut from the unwashed and washed nets of the different treatments were tested and field collected pyrethroid-resistant mosquitoes were used. The assay took place in the laboratory by releasing 100 non-blood-fed *An. gambiae* s.l. females at 7:00 p.m. in the biggest compartment of the tunnel made of glass, and a guinea pig was placed in the smallest confined compartment as a positive attractant. The two compartments were separated with a cardboard frame which contained a net piece (25 cm × 25 cm) holed by nine holes each 1 cm diameter. Dead, living, blood-fed and non-blood fed mosquitoes were removed from the compartments on the following day at 08:00 a.m. Living mosquitoes were observed for 72 h for delayed mortality. Treated nets washed and unwashed were compared to a control untreated one. The efficacy of each LN was measured in terms of: (i) blood-feeding inhibition (the reduction in blood-feeding compared with that from the control tunnel); (ii) mortality: proportion of mosquitoes collected dead after contact and which died 72 h after removed from the tunnel. Tunnel design can be found in the WHO Guidelines for laboratory and field-testing of long-lasting nets [29].

## Results

### Experimental huts

From August to October 2014, a total of 4867 *An. gambiae* s.l. were collected from the 6 experimental huts during the trial (Table 1). A significant reduction in mosquito numbers was observed in unwashed treated (including the chlorfenapyr dipped nets) than untreated nets (One-Way ANOVA, P < 0.05, Table 1). However, after washed, huts with treated nets collected more mosquitoes than the untreated control hut, suggesting that the deterrence effect had diminished with washing. Overall huts with washed treated nets had significantly more mosquitoes than their unwashed counterparts (One-Way ANOVA, P < 0.05) while no difference was seen between unwashed treated nets (One-Way ANOVA, P > 0.05, Table 1).

**Table 1:** Experimental hut trial results against pyrethroid resistant *Anopheles gambiae* s.l.

	Untreated	Chlorfenapyr 200 mg/m^2^	Interceptor LN (standard)		Interceptor G 2	
			Unwashed	Washed 20 times	Unwashed	Washed 20 times
Total females caught	853	643	519	1198	626	1028
Deterrence (%)		25^b^	39^b^	0^a^	27^b^	0^a^
Total in verandah trap	264	228	216	446	272	482
Exiting % (95% CI)	30^a^ (24.4–35.5)	44.50^a^ (38.5–50.4)	51.13^b^ (44.0–58.2)	37.63^a^ (34.0–41.2)	54.50^b^ (46.9–64.0)	51.50^b^ (36.6–66.3)
(%) Insecticide induce exiting	–	33^a^	41^b^	20^a^	45^b^	42^b^
Total females blood-fed	553	379	310	770	335	531
Blood-feeding % (95% CI)	68^a^ (53.7–82.7)	54^a^ (45.8–61.1)	52^a^ (45.6–58.3)	59^a^ (52.7–67.0)	38^b^ (26.2–50.2)	45^a^ (36.4–53.5)
Personal protection %		22^a^	24^a^	14^a^	44^a^	34^a^

^a,b^ Values along each line sharing the same letter superscript are not significantly different at the 5%

Induced exophily was minimal with all treated nets. Chlorfenapyr dipped net and Interceptor^®^ 20 times washed standard nets posited no difference in mosquito exiting rates compared to huts equipped with an untreated net (One-Way ANOVA, P > 0.05, Table 1). Interceptor^®^ G2 unwashed and 20 times washed nets had significantly more mosquitoes exiting into the verandah than the control, chlorfenapyr dipped net and the 20 times washed standard Interceptor^®^ net (One-Way ANOVA, P < 0.05, Table 1).

**Fig. 1:**
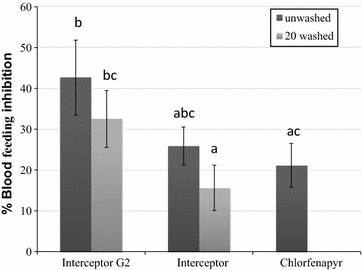
Blood feeding inhibition rates of *Anopheles gambiae* s.l. collected in experimental huts with treatments relative to control. Means comparison values for histograms sharing the *same letter* label are not significantly different (P > 0.05). *Error bars* represent 95% confidence intervals

Blood-feeding inhibition of *An. gambiae* s.l. populations relative to control (untreated net) was not evident with chlorfenapyr net 200 mg/m^2^, the standard Interceptor^®^ net (both washed and unwashed) and the Interceptor^®^ G2 net washed 20 times (Fig. 1). Interceptor^®^ G2 unwashed, resulted in blood-feeding levels significantly lower than the untreated net (Mann–Whitney, 42 vs 68%, P = 0.001; 38 vs 68%, P = 0.001) (Table 1).

Personal protection against mosquito biting with Interceptor^®^ G2 unwashed nets (44%) as well as washed (38%) was higher than standard Interceptor^®^ (unwashed and washed) and chlorfenapyr when applied to nets alone (Table 1).

**Fig. 2:**
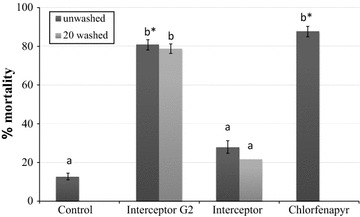
Mortality rates of *An. gambiae* s.l. collected in experimental huts with treatments versus untreated control. Means separation values for histograms sharing the *same letter* label are not significantly different (P > 0.05). *Error bars* represent 95% confidence intervals. *Asterisk* denoted highly significant

Interceptor^®^ G2 unwashed and washed 20 times killed ~80% of *An. gambiae* that entered the huts (Fig. 2). The dipped chlorfenapyr net killed up to 90%, but mortality induced by the standard Interceptor^®^ unwashed and washed 20 times was ~20% and was not significantly different from the untreated control net (One-Way ANOVA, P > 0.05, Fig. 2). No significant difference between the unwashed and the 20 times washed Interceptor^®^ G2 was observed, suggesting that these nets preserved their protective effect even after being washed 20 times.

### Cone tests in situ

**Fig. 3:**
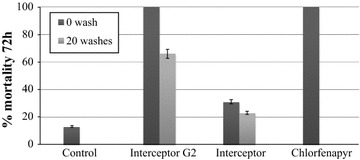
Mortality of *An. gambiae* adult females that were field collected as larvae from Vallée du Kou. Cone test bioassays were conducted in situ

Susceptibility tests using WHO cones confirmed full susceptibility of *An. gambiae* Kisumu to all treatments after 30 min contact (Fig. 3). Mortality to Interceptor^®^ G2 washed, unwashed and to the dipped chlorfenapyr nets to field collected mosquitoes at 72 h observation ranged from 75 to 95% and was significantly greater than that of the standard Interceptor^®^ net (One-Way ANOVA, P < 0.05). No significant difference between unwashed and washed Interceptor^®^ G2 nets was observed (Mann–Whitney, P = 0.917, Fig. 3).

### Tunnel test

The Interceptor^®^ G2 net (washed and unwashed) and the dipped chlorfenapyr net significantly killed (~80 to 95%) more field collected mosquitoes in tunnel tests than the standard Interceptor^®^ net (Table 2). Mortality to the standard Interceptor^®^ net (washed and unwashed) was not different from that of the untreated control net (P > 0.05). Blood-feeding inhibition was higher with the Interceptor^®^ G2 (unwashed and washed) than the standard Interceptor^®^ unwashed and washed nets (Table 2). Unwashed and 20 times washed Interceptor^®^ G2 performed equally well in terms of mortality and blood-feeding inhibition (Table 2).

**Table 2:** Tunnel test results with pyrethroid resistant *of Anopheles gambiae* s.l. adult females that were field collected as larvae from Vallée du Kou

	Untreated	Chlorfenapyr 200 mg/m^2^	Interceptor LN (standard)		Interceptor G 2	
			Unwashed	Washed 20 times	Unwashed	Washed 20 times
Number tested	204	220	243	203	246	187
Mortality (%)	5^a^	88^b^	26^a^	6^a^	94^b^	81^b^
95% CI	0–11.04	85.69–90.98	14.82–36.96	0–14.48	86.82–100	59.52–100
Penetration (%)	59^b^	26^a^	45^b^	50^b^	16^a^	18^a^
95% CI	39.88–77.11	14.72–36.27	35.20–54.79	40.68–58.31	1.20–20.79	16.52–18.47
Blood-fed (%)	69	21	44	35	8	7
95% CI	62.19–75.86	1.95–40.27	40.79–46.93	26.18–42.90	0–18.19	0–16.48
Blood-feeding inhibition (%)	–	69^b^	36^a^	50^a^	87^b^	90^b^

^a,b^ Values along each line sharing the same letter superscript are not significantly different at the 5% level

### Chemical analysis

**Fig. 4:**
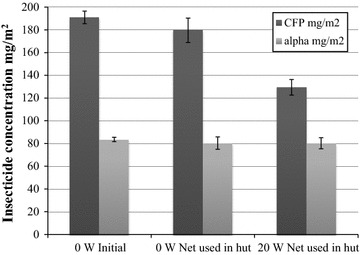
Insecticide concentration in a.i. mg/m^2^ of Interceptor G2 on initial and used nets

The chemical contents in the treated nets before and after washing is summarized in Fig. 4. Insecticide concentrations (both chlorfenapyr and alpha-cypermethrin) in a subsample of the initial nets and those actually used in the huts was not significantly different from one another (One-Way ANOVA, P > 0.05, Fig. 4). Initial chlorfenapyr loading on nets were reduced by 61 mg/m^2^ or ~32% when washed 20 times and used in huts. Additionally, chlorfenapyr content from initial loading on nets was reduced by 11 mg/m^2^ or ~6% when used in huts unwashed. The initial alpha-cypermethrin content was reduced by 4 mg/m^2^ ~5% in both cases.

## Discussion

Country-wide surveys in Burkina Faso have documented increasing levels of insecticide resistance in malaria vectors with a dramatic rise in the frequency of the *kdr* 1014F allele over the last decade, and the occurrence of the resistant *Ace*-*1* ^*R*^ 119S allele in both *An. coluzzii* and *An. gambiae* [13–16, 30, 31]. The aim of the study was to measure the efficacy of Interceptor^®^ G2 nets, treated with a mixture of alpha-cypermethrin and chlorfenapyr, in a such a complex environment where malaria vectors are highly resistant to pyrethroids and exhibit multiple mechanisms of resistance [16, 18]. The aim of the current study was to determine if the Interceptor^®^G2 nets could sustain the same level of efficacy after being washed 20 times as recommended by the WHOPES [32]. According to WHO, LNs must be effective after 20 washing.

As such, we evaluated the potential of the nets to: (i) provide individual protection against mosquito biting; and (ii) restore the effective control of pyrethroid resistant *An. gambiae* s.l. populations at Vallée du Kou 7 (VK7), where resistance is now well-established to pyrethroids.

Several studies have recently questioned the efficacy of pyrethroid treated nets where rapidly developing insecticide resistance is being observed. In such a context, there is an urgent need for managing insecticide resistance with new tools that can complement existing ones [25, 33]. Mixtures of carbosulfan and a pyrethroid were evaluated on mosquito nets, but results of these previous studies were not advanced due to mammalian toxicity issues associated with the carbamate [34].

In this study, superior performances of Interceptor^®^ G2 nets were achieved compared to the standard WHOPES recommended Interceptor^®^ in experimental hut trials. The significance of this study can be emphasized enough, where Interceptor^®^ G2 not only sustained higher mortality rates of wild mosquitoes compared to the WHOPES recommended standard Interceptor^®^ net, but Interceptor^®^ G2 effectively controlled well-documented, highly pyrethroid resistant mosquito populations. Although concentrations of alpha-cypermethrin (200 mg/m^2^) in the standard Interceptor^®^ net is higher than that of Interceptor^®^ G2 (100 mg/m^2^), the latter nets have an improved protective effect in terms of biting reduction. The combinational effects of two discrete and completely different modes of action to a single vector target have only begun to be investigated. Although the mode of action for chlorfenapyr is known to be slower, owing to the mitigation of protons across the inner mitochondrial membranes [36], it remains relatively unclear how intoxication and/or conversation via metabolic detoxification (by mosquitoes) in the presence of another insecticide like alpha-cypermethrin is influencing the observed behaviour. Another finding in this study demonstrated that there was a significant increase of exiting rate with Interceptor^®^ G2 washed 20 times compared to standard Interceptor^®^ washed 20 times. These results demonstrate that combining alpha-cypermethrin with chlorfenapyr on the same net afford benefits from the unique properties of each insecticide: the protective (excito-repellent) effect of the alpha-cypermethrin and the enhanced mortality to resistant mosquitoes through a completely novel mode of action in chlorfenapyr. The long-lasting formulation which combines these dual modes of action on a single net that is wash resistant and adheres to the WHOPES criteria for durability is profound, and underscores one of the more daunting reasons other modes of actions have not been routinely applied to LNs—namely the incompatibility of formulation(s), limits to physical-chemistry and solubility needed to sustain both mortality and wash resistance on or in nets. The protective effect of the pyrethroid and the killing effect of the chlorfenapyr against pyrethroid resistant *Anopheline* and *Culicine* mosquitoes confirm the potential of the mixture of pyrethroid and a pyrrole on the same net as an alternative ITN treatment [33]. In experimental huts the Interceptor^®^ G2 provided high mortality against wild pyrethroid resistant *An. gambiae* s.l. Interestingly, the mortality with Interceptor^®^ G2 unwashed was not significantly different from that of the 20 times washed of the same nets. Results from the tunnel test also confirm the superior killing effect and blood-feeding inhibition of the unwashed and washed Interceptor^®^ G2 nets compared to the standard Interceptor^®^ nets.

Among the more significant findings recently reported in literature, it is clear that testing modality for non-neurotoxic compounds like chlorfenapyr can be highly influential [35]. In the present study, it was observed that standard WHOPES cone tests, which principally measure the biological impact of a chemical on mosquitoes through forced direct exposures, posited high mortality to adult mosquitoes and provides evidence that the combination of chlorfenapyr with alpha-cypermethrin on a net has a real potential to control pyrethroid resistant mosquitoes. This should be considered carefully in all future studies, as cone bioassays can be problematic for mosquito exposures to a physiological toxin like chlorfenapyr, as identified by Oxborough et al. [35].

The active ingredient content recovered by analytical determination for chlorfenapyr exhibited only moderate loss of active ingredient over 20 washes, and clearly had no observable effect on the level of control chlorfenapyr exacted on mosquitoes in this study. The loss of Interceptor^®^ G2 alpha-cypermethrin active ingredient content was proportionately reduced compared to active alpha-cypermethrin ingredient loss (from washing) reported from WHOPES recommended Interceptor^®^ nets. The chlorfenapyr control (chlorfenapyr-dipped net) was effective in killing pyrethroid resistant mosquitoes. Interceptor^®^ G2 nets afford protection that cannot be realized with Interceptor^®^ nets. Because higher levels of mortality were observed from exposures to chlorfenapyr control and Interceptor^®^ G2 nets with chlorfenapyr, the two positive controls clarify the relative contribution of chlorfenapyr compared to that which alpha-cypermethrin alone can contribute. Alpha-cypermethrin has lost its killing effect at this location, but the combination of both in Interceptor^®^ G2 were effective against mosquitoes even given the pervasive nature of resistant alleles at the VK7 site. The nets evaluated in this study clearly demonstrated improved performance of Interceptor^®^ G2 with a good personal protective rate, and an improved ability to kill pyrethroid resistant mosquito populations.

Under the present experimental conditions, Interceptor^®^ G2 LN outperformed the WHOPES recommended Interceptor^®^ LN washed 20 times and hence meets the WHO criteria for LNs. Our work suggests that long-lasting mixture of chlorfenapyr and alpha-cypermethrin on nets has a real potential in controlling pyrethroid resistant mosquitoes in Africa and should be urgently developed and used as a pyrethroid IRM tool in areas relevant to its need. It also marks the first LN with two discrete modes of action (two adulticides) which are complementary to each other and afford improved user protection while maintaining safety and utility.
